# Indications for the Administration of Digitalis

**Published:** 1882-02

**Authors:** 


### INDICATIONS FOR THE ADMINISTRATION OF DIGITALIS.

In the *Deutsche med Zeitschrift*, No. 23, 1881, Prof. Leyden states his views in regard to the use of digitalis. He thinks the drug is of advantage in acute febrile diseases, in hæmoptysis and other hemorrhages, and of course in cardiac diseases. As an antipyretic, however, it is not a very safe agent, on account of the danger of sudden collapse. As regards its employment in heart disease, he thinks it difficult to lay down any generally applicable rules. Every case should be judged on its own individual merits. In mitral and and aortic affections there is a special indication for its use when there is diminished arterial tension, with dyspnœa and scanty secretion of urine. He finds it best in such cases to give only small doses, one-quarter or one-fifth of a grain daily. If these doses fail, it remains an open question whether or not it be advisable to give more. In hypertrophy and dilatation of the left ventricle, with co-existing pulmonary disturbance, digitalis is a highly uncertain remedy. As a diuretic its action is feeble, succeeding only when the action of the left ventricle is feeble. Leyden prefers the tincture and infusion of digitalis to other preparations of the drug. The best substitute for digitalis he thinks is squills. Both drugs may be given in combination, or, better still, the exhibition of digitalis may be followed by that of squills.—*Med. Record.*

